# Effect of Dietary Intake of Avocado Oil and Olive Oil on Biochemical Markers of Liver Function in Sucrose-Fed Rats

**DOI:** 10.1155/2014/595479

**Published:** 2014-04-17

**Authors:** Octavio Carvajal-Zarrabal, Cirilo Nolasco-Hipolito, Ma. Guadalupe Aguilar-Uscanga, Guadalupe Melo Santiesteban, Patricia M. Hayward-Jones, Dulce Ma. Barradas-Dermitz

**Affiliations:** ^1^Biochemical and Nutrition Chemistry Area, University of Veracruz, SS Juan Pablo II s/n, 94294 Boca del Río, VER., Mexico; ^2^Department of Molecular Biology, Faculty of Resource Science and Technology, Universiti Malaysia Sarawak, Kota Samarahan, 94300 Sarawak, Malaysia; ^3^Food Research and Development Unit, Veracruz Institute of Technology, Calzada M. A. de Quevedo 2779, 91860, Veracruz, VER., Mexico; ^4^Pathology Laboratory, Institute of Forensic Medicine, University of Veracruz, SS Juan Pablo II s/n, 94294, Boca del Río, VER., Mexico; ^5^Biological-Chemistry Area, Veracruz Institute of Technology, Calzada M. A. de Quevedo 2779, 91860, Veracruz, VER., Mexico

## Abstract

Metabolic changes, along with cardiovascular and hepatic factors, are associated with the development of diseases such as diabetes, dyslipidemia, and obesity. We evaluated the effect of avocado oil supplementation (centrifuged and solvent extracted), compared with olive oil, upon the hepatic function in sucrose-fed rats. Twenty-five rats were divided into five groups: control (basal diet), a sucrose-fed group (basal diet plus 30% sucrose solution), and three other groups (S-OO, S-AOC, and S-AOS, indicating basal diet plus 30% sucrose solution plus olive oil OO, avocado oil extracted by centrifugation AOC or using solvent AOS, resp.). Glucose, total cholesterol, triglycerides, total protein, albumin, globulin, direct bilirubin, glutamic pyruvic transaminase, glutamic oxaloacetic transaminase, alkaline phosphatase, cholinesterase, and **α**-amylase concentrations were determined and avocado oil effect on them was studied. In some cases the induced metabolic alteration significantly affected total protein and bilirubin levels and also had a highly significant effect on **α**-amylase levels. AOC and AOS exhibited effects similar to those of olive oil, according to the nonsignificant difference in fatty acid profile observed by other authors. Avocado oil consumption could be beneficial in the control of altered metabolic profile illnesses as it presents effects on hepatic function biochemical markers similar to olive oil.

## 1. Introduction

The incidence of metabolic syndrome in Mexico is one of the highest in the world, so much that it has become a public health problem. However, little has been done to prevent the factors responsible for this. Epidemiological studies in our country highlight the need to strengthen strategies for its detection, control, and treatment. It involves a set of three or more alterations such as overweight or obesity and disturbance in glucose metabolism and insulin, along with hypertension, dyslipidemia, and other abnormalities of importance that are related to its development and are grouped in different profiles, such as liver, pancreatic, and cardiovascular functions [1–4]. The incidence is about 25% in the general population and there are no differences between men and women, although it varies according to genetic factors [5, 6]. Reports in the scientific literature show the benefits that the Mediterranean diet and olive oil have on health [7, 8]. These have aroused interest in studying oils rich in monounsaturated fatty acids, especially avocado oil and its effect on manifestations of health disorders in metabolic abnormality. In nonpharmacological treatment, the consumption of monounsaturated fatty acids such as oleic acid, found in different types of oils like olive and avocado, is recommended. The avocado fruit is a rich potential source of oil, mostly monounsaturated [9], and a good source of linoleic acid [10]. It also contains high levels of antioxidants including polyphenols, proanthocyanidins, tocopherols, and carotenoids, which have shown positive outcomes in health. It has also been established that soluble components of avocado oil confer these properties. Studies in humans and animal models have showed that it helps to control weight, reduces the risk of diabetes [11], normalizes blood cholesterol levels [12], and is involved in liver metabolism [13]. In addition, the phytochemical components of avocado oil are also related to the disease manifestations associated with an altered metabolic profile, so, overall, it is expected that all the beneficial properties of avocado oil together will add up to positive health effects. The purpose of this study was to evaluate the effect of avocado oil supplementation, as compared to that of olive oil, upon biochemical markers and hepatic function associated with a condition induced in rats through the administration of sucrose in drinking water.

## 2. Materials and Methods

### 2.1. Avocado Oil Extraction

There are different technologies for extracting oil from avocados and they can affect its quality. The oil was obtained from Hass avocado purchased from a local market in the Port of Veracruz, Mexico. When edible maturity had been reached, the avocados were washed and peeled and the seed removed. Subsequently, the pulp was homogenized by adding tert-butylhydroquinone (TBHQ) at 0.1% (w/w).

#### 2.1.1. Oil Extraction by Centrifugation

The avocado pulp was mixed with water to achieve a 1 : 1 w/v and NaCl (7.5% w/w), the pH was adjusted to 5.5 with ascorbic acid and the mixture was homogenized in a blender (Black & Decker Model MX 150) at 1,300 rpm for 1 hour at 35°C. Subsequently, the oil was removed by centrifugation at 27000 rpm in a tubular continuous centrifuge (Cepa-Schnell, GLE Model NBS) fed at 2.8 L/min.

#### 2.1.2. Avocado Oil Extraction by Solvent

A homogenate was made with a portion of the avocado pulp and two parts of a mixture of hexane-isopropanol (2 : 3 v/v) in separate funnels, and the oil phase was collected. Subsequently, the solvent was removed in a rotary evaporator (Buchi R-215, Labortechnik AG, Switzerland) at 30°C and 500 mmHg pressure. The remaining solvent was removed by entrainment with nitrogen gas and then the oil was exposed to high vacuum in a freeze dryer for 24 h. Thereafter the oil was stored in refrigeration and protected from light until use.

### 2.2. Animals and Treatments

In this experiment 25 male Sprague-Dawley weaned rats (3 weeks old and weighing 240 ± 16 g) were purchased from Teklad, Co. (Mexico City), and caged individually in stainless steel boxes in a room with controlled temperature (25°C) and a light-dark cycle of 12 hours. The experimental protocol for the management of experimental animals was approved by the animal ethics committee, Biochemical and Nutrition Chemistry Area, University of Veracruz. The basal diet was prepared according to the American Institute of Nutrition [14] as shown in Table 1. A mixture of corn-canola oil (7.5 g/100 g diet) was used as a source of dietary fat (Patrona from the local market). The experimental diet was prepared based on the composition of the basal diet plus oil (7.5% w/w), olive oil (Carbonell), and avocado oil extracted by centrifugation or solvent, respectively. Diets were prepared once a week and kept in powder form 4°C until use. As part of this study, the fatty acid composition of the oils used in preparing diets was analyzed and it was found that all the oils had a rather similar composition, mainly oleic and linoleic acids (Table 2).

The animals were divided into two groups: a control group (CG, *n* = 5) receiving a basal diet and a sucrose-fed group (S, *n* = 20) which received the basal diet plus 30% sucrose solution as drinking water. The animals had free access to food and water for 16 weeks and food intake was measured daily. At the end of this period, the diet was withdrawn for at least 4 hours and the manifestation of the metabolic characteristics was checked by first determining body weight, then serum glucose, triglycerides, and cholesterol levels by cardiac puncture.

Once the sucrose-fed model had been obtained, the S animals were divided into four groups of five rats each. One group was maintained on the basal diet (the sucrose-fed group, S); three groups of rats designated, as S-OO, S-AOC, and S-AOS, respectively, received an experimental diet containing 7.5% w/w oil (olive and avocado extracted by centrifugation or extracted with solvent) as the sole source of dietary fat. These four groups received the experimental diets and water with 30% sucrose solution for 4 weeks. The CG group continued to receive only the diet with corn-canola oil and no sucrose in the drinking water. Diets were prepared once a week and kept refrigerated until use. Tert-butylhydroquinone (TBHQ) at 0.02% was used to prevent fatty acid oxidation. At the end of the experiment, the diet was withdrawn and the fasting animals were sacrificed through decapitation. Serum glucose, cholesterol, triglyceride, and phospholipid levels were determined. All animals were sacrificed and the organs were extracted for further analysis.

### 2.3. Assays

Glucose was determined with the glucose oxidase method. Total cholesterol, triglycerides (TG), total protein, albumin, globulin, direct bilirubin, glutamic pyruvic transaminase (GPT), glutamic oxaloacetic transaminase (GOT), alkaline phosphatase (ALP), cholinesterase, and *α*-amylase were determined with an automated analyzer (RA 1000 XT, Bayer Technicon) through enzymatic colorimetric methods using commercial kits obtained from Bayer and BioMerieux. The fatty acid profile of vegetable oils was determined through gas chromatography (Hewlett Packard 5890, Palo Alto, CA.) with pentadecanoic acid as internal standard. All the chemicals used were of analytical grade.

### 2.4. Statistical Analysis

The data are expressed as the mean ± standard deviation (*x* ± SD). Statistical significance was determined with analysis of variance procedures, with a post hoc Tukey multiple-range test for comparison of means (*P* < 0.05).

## 3. Results

### 3.1. Metabolic Characteristics of Rats in the Control and Sucrose-Fed Group

Table 3 shows growth variables, food and caloric intake, liquid consumption, and biochemical markers to assess rats in the control group (CG) and sucrose-fed rats (S). At 16 weeks, a significant increase (*P* < 0.05) in final body weight and body weight gain was observed in the S group as compared to the CG group. The food intake in rats in the CG group was significantly higher (*P* < 0.01) than in the S group. On the other hand, the S group showed a daily liquid intake significantly higher (*P* < 0.05) as compared with the CG group. However, when the daily liquid intake per 100 g in weight was compared between CG and S group, this was not significant. The caloric equivalent produced by liquid intake was 10.8 ± 1.7 kcal in the S group; the CG group did not have any energy intake because this group received only purified drinking water. Triglyceride levels in the S group were significantly greater (*P* < 0.01) than in the CG group; however, no significantly different results were found in any group for either glucose or cholesterol levels.

### 3.2. Effect of Dietary Oils on Liver Function Biochemical Markers

**Figure 1:**
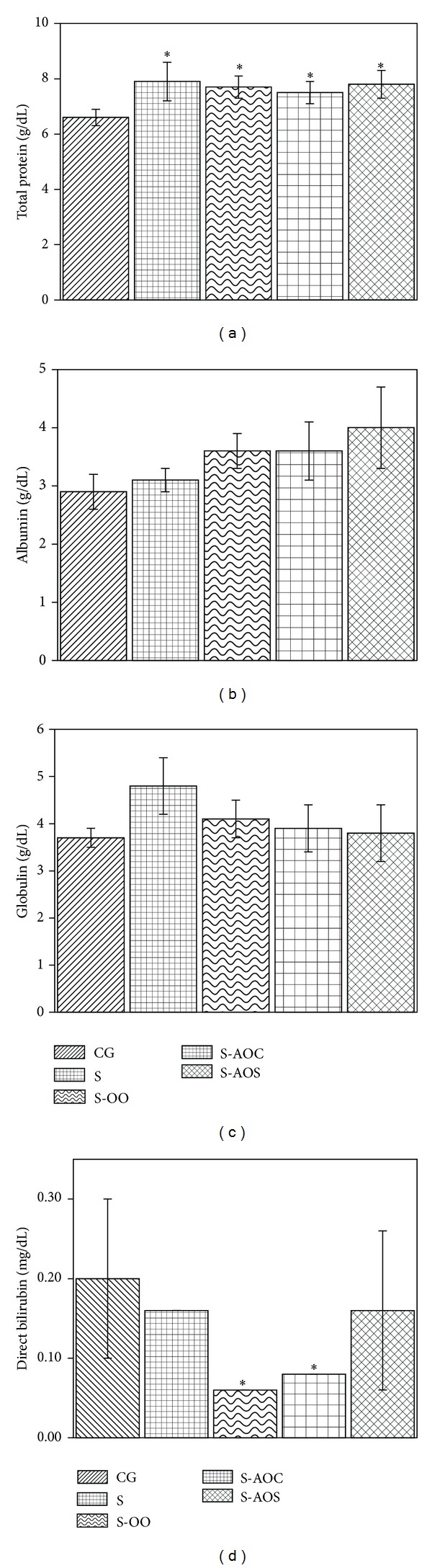
Blood serum profile levels of liver function markers in rats fed with different oil diets: a) total protein; b) albumin; c) globulin; d) direct bilirubin. Corn-canola diet plus 30% sucrose in drinking water (S group, *n* = 5); olive oil diet plus 30% sucrose in drinking water (S-OO group, *n* = 5); avocado oil diet extracted by centrifugation plus 30% sucrose in drinking water (S-AOC group, *n* = 5); avocado oil diet extracted by solvent plus 30% sucrose in drinking water (S-AOS group, *n* = 5). Values are mean ± SD. *P < 0.05; **P < 0.01 versus corresponding data in CG group.

The effect of olive and avocado oils on liver function indicators is shown in Figure 1. S, S-OO, S-AOC, and S-AOS study groups showed changes in total protein levels, all of them significantly higher (*P* < 0.05) than control group (CG). Additionally, direct bilirubin levels in S-OO and S-AOC groups decreased (*P* < 0.05) in relation to CG and S groups, but not for the S-AOS group, where a nonsignificant decrease was observed. Albumin and globulin levels in S-OO, S-AOC, and S-AOS study groups were not found to be significantly different either from S or CG groups.

### 3.3. Effect of Dietary Oils on Pancreatic Function Biochemical Markers

**Figure 2:**
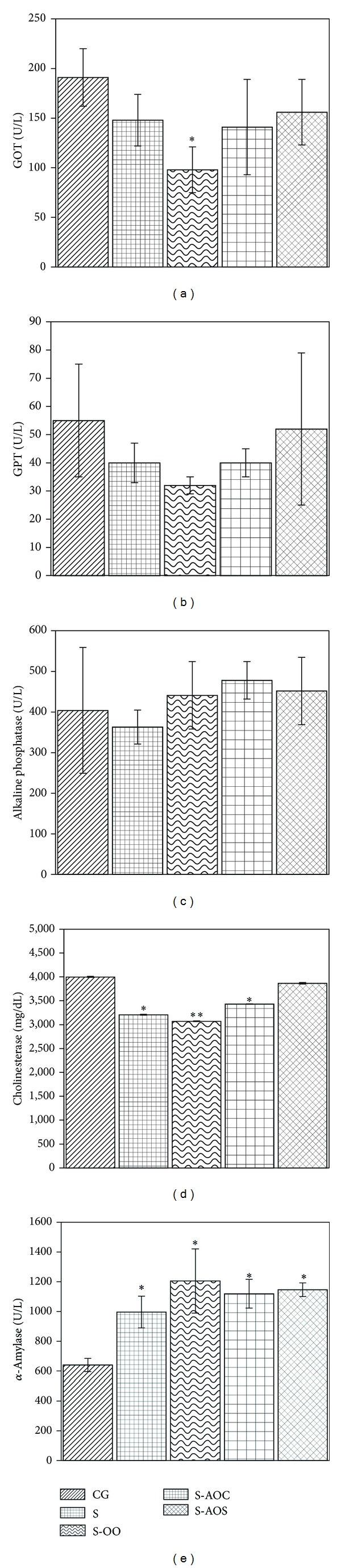
Blood serum profile levels of liver function markers in rats fed with different oil diets: a) GOT; b) GPT; c) Alkaline phosphatase; d) Cholinesterase; e) *α*-amylase. Corn-canola diet (CG group, *n* = 5); corn-canola diet plus 30% sucrose in drinking water (S group, *n* = 5); olive oil diet plus 30% sucrose in drinking water (S-OO group, *n* = 5); avocado oil diet extracted by centrifugation plus 30% sucrose in drinking water (S-AOC group, *n* = 5); avocado oil diet extracted by solvent plus 30% sucrose in drinking water (S-AOS group, *n* = 5). GOT: glutamic oxaloacetic transaminase; GPT: glutamic pyruvic transaminase. Values are mean ± SD. *P < 0.05; **P < 0.01 versus corresponding data in CG group.

The effect of dietary olive and avocado oils on pancreatic function indicators is shown in Figure 2. Levels of glutamic oxaloacetic transaminase (GOT) in S-AOC and S-AOS groups were similar and not significantly different than either the S or CG group. Significantly lower levels were observed in the S-OO group (*P* < 0.05) in comparison with all the groups in the study. In the cases of glutamic pyruvic transaminase (GPT) and alkaline phosphatase (ALP), no significant results were observed among the study groups. The S-AOS group showed similar, nonsignificant values for cholinesterase in comparison with CG. In contrast, S and S-AOC groups both presented significantly lower results (*P* < 0.05), but in the case of S-OO group these levels decreased in a highly significant manner (*P* < 0.01) when compared to CG and S-AOS. Levels of *α*-amylase were all similar for S, S-OO, S-AOC, and S-AOS groups; however, when compared to control group CG, the results were all very significantly higher (*P* < 0.01).

## 4. Discussion

Diabetes, dyslipidemia, and obesity are risk factors with a great impact on the development of diseases associated with an altered metabolic profile or metabolic syndrome. Liver and heart factors also play an important role.

Within this framework, in the present study, significant differences were found for S group as compared to the CG group in final body weight and weight gain, which were significantly higher (6 and 11% resp.), although food intake was significantly lower (54%). These results are consistent with those reported in other studies where metabolic changes were induced by the administration of a sucrose-rich diet in addition to an experimental diet causing changes in the biochemical indicators measured [15, 16]. In relation to serum biochemical indicators associated with the development of metabolic abnormality, it was found that glucose and cholesterol concentrations in S group rats were similar to those in the CG group and not significant. Reaven and Chang [17] have suggested that this is due to hyperinsulinemia developed in metabolic abnormalities which maintains normal levels of blood glucose. TG levels were significantly higher (56%) in S group rats (a 2.3-fold increase). Other studies have found similar results [18, 19]; Piatti et al. [20] reported the association in healthy patients between sudden TG elevation and insulin resistance and suggested that the increase in blood TG* in vivo* inhibits glucose utilization and oxidation stimulated by insulin action in the peripheral tissues. One way to explain the blood TG elevation might be to consider a possible increase in the reesterification of fatty acids from the liver as a result of fructose metabolism as reported by Bezerra et al. [18]; this monosaccharide stems from sucrose hydrolysis and, in the liver, fatty acids are mainly used for the high-density lipoprotein (HDL) and TG synthesis, which in turn raise their serum levels.

Sucrose intake did not wield a significant influence on total protein levels as values encountered in sucrose-fed study group and in those fed with olive oil and avocado oil extracted by centrifugation or by solvents were statistically similar (7.9, 7.7, 7.5, and 7.8 g/dL, resp.) for S, S-OO, S-AOC, and S-AOS groups. Nevertheless, total protein values for the sucrose-fed group (S), as well as for S-OO, S-AOC, and S-AOS groups, increased significantly (16, 14, 12, and 15%, resp.) compared to control (CG). This result suggests that avocado oil exerts an effect similar to that of olive oil on liver synthesis of total proteins which are used as markers for liver damage; a rise in these proteins is associated with the development of a nonalcoholic liver pathology, such as fatty liver, linked to metabolic syndrome [21].

Direct bilirubin indicates a loss in hepatocyte function; these levels decreased significantly in S-OO and S-AOC groups in this study compared to control (CG), sucrose-fed group (S), and the group fed with avocado oil extracted by solvent (70, 63, 63% and 60, 50, 50%, resp.). No significant differences were found for albumin and globulin levels in any group in the study. However, albumin levels in the groups fed with avocado oil extracted by centrifugation or solvent were slightly greater, although not significantly so, compared to control (3.6, 4.0 versus 2.9 g/dL, resp.). This indicates that albumin blood levels (a liver damage marker) are not brought back to normal levels by this oil, possibly suggesting that avocado oil composition as a result of the extraction method could have an influence on the level of regulation of this marker.

On the other hand, globulin levels in S group increased (23%), although not significantly, when compared to control. In other groups, S-OO, S-AOC, and S-AOS, globulin levels exhibited a nonsignificant decrease (15, 19, and 21%, resp.) compared to the sucrose-fed group (S) but were slightly higher, again not significantly so (10, 5, and 3%, resp.) than control (CG). These results indicate a return to normal globulin levels as a result of olive and avocado oil consumption. This could have induced a reversion to the damage caused to the liver parenchyma through steatosis, given that in this pathology blood globulin levels remain high.

It was also found that albumin/globulin ratio values (RAG) of rats fed olive oil and avocado oil extracted by centrifugation or by solvents were higher than the sucrose-fed group (0.9, 0.9, and 1.0 versus 0.7, resp.). The results for this marker could suggest a regeneration of liver tissue, as RAG values of the aforementioned study groups were close to 1, similar to the CG value. S group, on the other hand, had quite a low value for this ratio which suggests liver damage.

Mention should be made that to date no reports have appeared in the scientific literature about the effect of avocado oil on the basic profile of protein synthesized by the liver which are used as liver damage markers, specifically cirrhosis of the liver and fatty liver, thus warranting the determination of these values in the present study.

As for control group (CG), GOT and GPT values decreased in the rest of the groups (S, S-OO, S-AOC and S-AOS). An increase, not a decrease, in these enzymes is indicative of liver failure. The effect of administering a diet rich in carbohydrates (30% sucrose), known to contribute to metabolic syndrome in murine models through oxidative stress, or through the activation of adipocyte-specific genes (lipogenesis), among other effects, and in consequence to hepatic metabolic changes [22–24] was not observed in this case related to an increase in GOT and GPT values.

A significant decrease in GOT was observed in the S-OO group. Both olive and avocado oils are recognized as oils with a high percentage of unsaturated fatty acids and a low percentage of saturated [25, 26], (84, 88 and 17, 12%, resp.) in murine models, different transcriptomic responses between diets based on different long-chain polyunsaturated fatty acids have been observed. Furthermore, stereochemistry influences differential responses as seen with linoleic acid isomers [23].

Sucrose intake significantly decreased cholinesterase levels in the sucrose-fed group (S) by 20% compared to control. Olive oil did not improve this situation at all and in fact the results of the S-OO group were significantly even lower than S. Avocado oil extracted by solvent brought cholinesterase levels back to normal, similar to those of control (3866 versus 3999 mg/dL resp.), but, for the S-AOC group, results remained at the same level statistically as the sucrose-fed group (S).

A decrease in cholinesterase levels can be indicative of hepatic disease. As far as can be ascertained, the different responses of olive and avocado oil observed here have not been reported before. A possible explanation could be based on the specific chemical composition of each oil type and their capacity to overcome the diet induced metabolic alterations. The unsaponifiable fraction of fatty oils contains more than 100 components. A specific example is the case of olive oil whose fraction contains more than 300 components [27]. It has been demonstrated that these minor components also exert effects on the metabolic pathways and according to Osada, 2013, the term “monounsaturated fatty acid-enriched oil” including oils such as olive and other oils, based on the high percentage of oleic acid, “no longer appears appropriate for describing their biological properties since they have different unsaponifiable composition and this fraction is highly active.”

For S, S-OO, S-AOC, and S-AOS groups in the study, *α*-amylase levels were found to be significantly higher compared to control (36, 47, 43, and 44%, resp.). This points to pancreas damage which was not reversed by the administration of dietary oils (S-OO, S-AOC, and S-AOS); in these groups, significantly higher *α*-amylase levels were found (1205, 1119, and 1146 versus 996 U/L, resp.) compared to S. To our knowledge, no data are available in the literature about the effect of avocado oil on the levels of this enzyme, even less where sucrose ingestion is concerned.

Sucrose intake caused no effect in ALP levels in S-OO, S-AOC, and S-AOS groups as these were similar and did not differ from control (CG) or sucrose-fed group (S). However, ALP levels in S-AOC and S-AOS groups were seen to increase (15 and 11%, resp.) in comparison with control and 24 and 20%, respectively, when compared to S. It has been reported that in cases of nonalcoholic fatty liver disease alkaline phosphatase levels increase and are a favorite marker for metabolic syndrome [28].

## 5. Conclusion

To sum up, these results indicate that sucrose intake affected total protein and bilirubin levels; the results of other markers did not show any evidence of liver damage, probably because the window for manifestation was very short. However, variations in the aforementioned marker levels indicate that the liver was affected and that the albumin/globulin ratio (RAG) under the influence of avocado oil administration revealed the beginning of a regeneration of liver function. On the other hand, neither the administration of olive oil nor avocado oil extracted by centrifugation or using solvent was able to attain normal *α*-amylase levels, indicating that anomalies in pancreatic function were not reversed. Avocado oil exhibits effects similar to those of olive oil, and no differences in biochemical markers were found between the two methods of avocado oil extraction. This finding is correlated to the nonsignificant difference observed in fatty acid profile of avocado oils obtained by the two aforementioned extraction methods, as reported by Ariza-Ortega et al. [29].

## Figures and Tables

**Table 1:** Composition of basal and experimental diets formulated according to AIN-76G.

Ingredients	Basal diet (g)
Cornstarch	65.8
Casein	44.0
Cellulose	4.0
Mineral Mix AING-76 G	8.0
Vitamin Mix AING-76 G	2.0
DL-Methionine	0.32
Tert-butylhydroquinone	0.02
Fat^†^	10.0

^†^Corn-canola, olive, or avocado oil. Experimental diets were formulated with basal diet plus oil: olive or avocado oil extracted either by centrifugation or solvent.

**Table 2:** Fatty acid composition of dietary oils (%).

Fatty acid	Corn	Canola	Olive	Avocado^c^	Avocado^s^
16:0	10.0	7.5	15.0	17.0	16.0
16:1	0.1	0.2	1.9	8.3	6.5
18:0	2.4	3.3	2.4	0.5	0.5
18:1	39.0	32.0	59.4	54.4	58.8
18:2	50.0	37.0	15.4	10.2	9.6
18:3	2.5	7.7	0.9	0.9	0.9

Values are expressed as mean of duplicate analysis. Avocado^c^: avocado oil extracted by centrifugation, Avocado^s^: avocado oil extracted by solvent.

**Table 3:** Growth, food and caloric intake, liquid consumption, and biochemical indicators in CG and S rats.

Parameters	Dietary groups	
	CG group	S group
Initial body weight (g)	239 ± 22	242 ± 24
Final body weight (g)	445 ± 53	470 ± 38*
Body weight gain (g)	206 ± 1.8	228 ± 2.0*
Food intake (g/d)	26.1 ± 1.3	14.3 ± 1.1**
Liquid consumption (mL/d)	46.3 ± 3.3	58.1 ± 3.4*
Liquid consumption (mL/d/100 g bw)	9.3 ± 1.4	10.5 ± 0.6
Equivalent in kcal in drinking water	0.00	10.8 ± 1.7**
Glucose (mg/dL)	114 ± 18	130 ± 11
Cholesterol (mg/dL)	104 ± 12	101 ± 12
Triglycerides (mg/dL)	79 ± 12	179 ± 35**

Values are mean ± SD. CG group, *n* = 5; S group, *n* = 20. *P < 0.05; **P < 0.01.
